# BioClocks UK: driving robust cycles of discovery to impact

**DOI:** 10.1098/rstb.2023.0345

**Published:** 2025-01-23

**Authors:** Hannah Rees, Nina M. Rzechorzek, Rebecca B. Hughes, Antony N. Dodd, James J. L. Hodge, Tyler J. Stevenson, Malcolm von Schantz, Robert J. Lucas, Sarah E. Reece, Charalambos P. Kyriacou, Andrew J. Millar

**Affiliations:** ^1^ Institute of Biological, Environmental & Rural Sciences (IBERS), Aberystwyth University, Plas Gogerddan, Aberystwyth SY23 3EE, UK; ^2^ Cell Biology Division, MRC Laboratory of Molecular Biology, Francis Crick Avenue, Cambridge Biomedical Campus, Cambridge CB2 0QH, UK; ^3^ Centre for Biological Timing and Division of Neuroscience, School of Biological Sciences, Faculty of Biology Medicine and Health, University of Manchester, Manchester M13 9PT, UK; ^4^ John Innes Centre, Norwich Research Park, Norwich NR4 7RU, UK; ^5^ School of Physiology, Pharmacology and Neuroscience, University of Bristol, Biomedical Sciences building, University Walk, Bristol BS8 1TD, UK; ^6^ School of Biodiversity, One Health and Veterinary Medicine, College of Medical, Veterinary and Life Sciences, University of Glasgow, Garscube Campus, Bearsden Road, Glasgow G61 1QH, UK; ^7^ Faculty of Health and Life Sciences, Northumbria University, Newcastle upon Tyne NE1 8ST, UK; ^8^ Institute of Ecology and Evolution & Institute of Immunology and Infection Research, School of Biological Sciences, University of Edinburgh EH9 3FL, UK; ^9^ Department of Genetics, Genomics and Cancer Sciences, University of Leicester, Leicester LE1 7RH, UK; ^10^ School of Biological Sciences and Centre for Engineering Biology, University of Edinburgh, Max Born Crescent, Edinburgh EH9 3BF, UK

**Keywords:** chronobiology, research network, research translation, impact, BioClocks UK, shift-work

## Abstract

Chronobiology is a multidisciplinary field that extends across the tree of life, transcends all scales of biological organization, and has huge translational potential. For the UK to harness the opportunities presented within applied chronobiology, we need to build our network outwards to reach stakeholders that can directly benefit from our discoveries. In this article, we discuss the importance of biological rhythms to our health, society, economy and environment, with a particular focus on circadian rhythms. We subsequently introduce the vision and objectives of BioClocks UK, a newly formed research network, whose mission is to stimulate researcher interactions and sustain discovery-impact cycles between chronobiologists, wider research communities and multiple industry sectors.

This article is part of the Theo Murphy meeting issue ‘Circadian rhythms in infection and immunity’.

## 1. Introduction

### (a) An introduction to the field of chronobiology

Chronobiology is the study of natural rhythmic processes governed by endogenous biological clocks. Biological clocks have been identified in all kingdoms of life, from bacteria to plants, fungi and animals, including humans. Alignment of the timing of biological processes with periodic environmental changes provides an adaptive evolutionary advantage [1,2]. The best-known biological rhythms are circadian rhythms, yet there are also non-circa-24-hour cycles, including ultradian rhythms lasting a few hours, the 12.4 h circatidal and semi-lunar (approx. 15 day) rhythms present in coastal organisms, or the menstrual cycles in several mammalian species. Longer infradian cycles include seasonal bird migration and overwintering of some insects and mammals. The influence of biological rhythms is particularly obvious when they are dysfunctional through disease or when organisms suffer desynchrony with their environment.

Early evidence for endogenous circadian rhythms includes 18th-century experiments describing circadian cycles of *Mimosa pudica* leaf position under constant darkness [3], temperature [4,5] and humidity [6,7]. The UK also has a rich heritage of chronobiology research. In 1845, John Davy measured daily rhythms of his own pulse and body temperature, using a purpose-built curved thermometer allowing readings with the bulb in his mouth [8]. In 1880, Charles and Francis Darwin observed ‘sleep movements’ of leaves, concluding that their periodicity was, ‘to a certain extent inherited’ [9]. In the 1950s, British-born biologist Colin Pittendrigh and German colleagues Erwin Bünning and Jürgen Aschoff laid the foundations for the field of chronobiology, coining the term ‘circadian’ to describe endogenous biological cycles with a period of approximately 24 hours (*circa* = approximately, *dies* = day) [10].

Over the past five decades, chronobiology research has expanded worldwide, strengthened by international collaboration and use of model organisms including insects, mammals, fungi, cyanobacteria and plants. Identification of the first circadian clock genes in *Drosophila* (1971) [11], *Neurospora* (1973) [12], mammals (1988) [13] and plants (1995) [14] unleashed extensive mechanistic and fundamental adaptive insights. Technological innovations in imaging and genetic modification allowed spatiotemporal characterization of clock function at tissue, cellular and subcellular levels. The subsequent ‘omics revolution’ enabled genome-wide studies of circadian regulation [15], and investigation of natural variation in circadian phenotypes [16–18]. There are attractive future opportunities for UK chronobiology at the interface between different chronobiological disciplines and through community coordination for translation of discovery research [19].

### (b) UK chronobiology today

**Figure 1.**
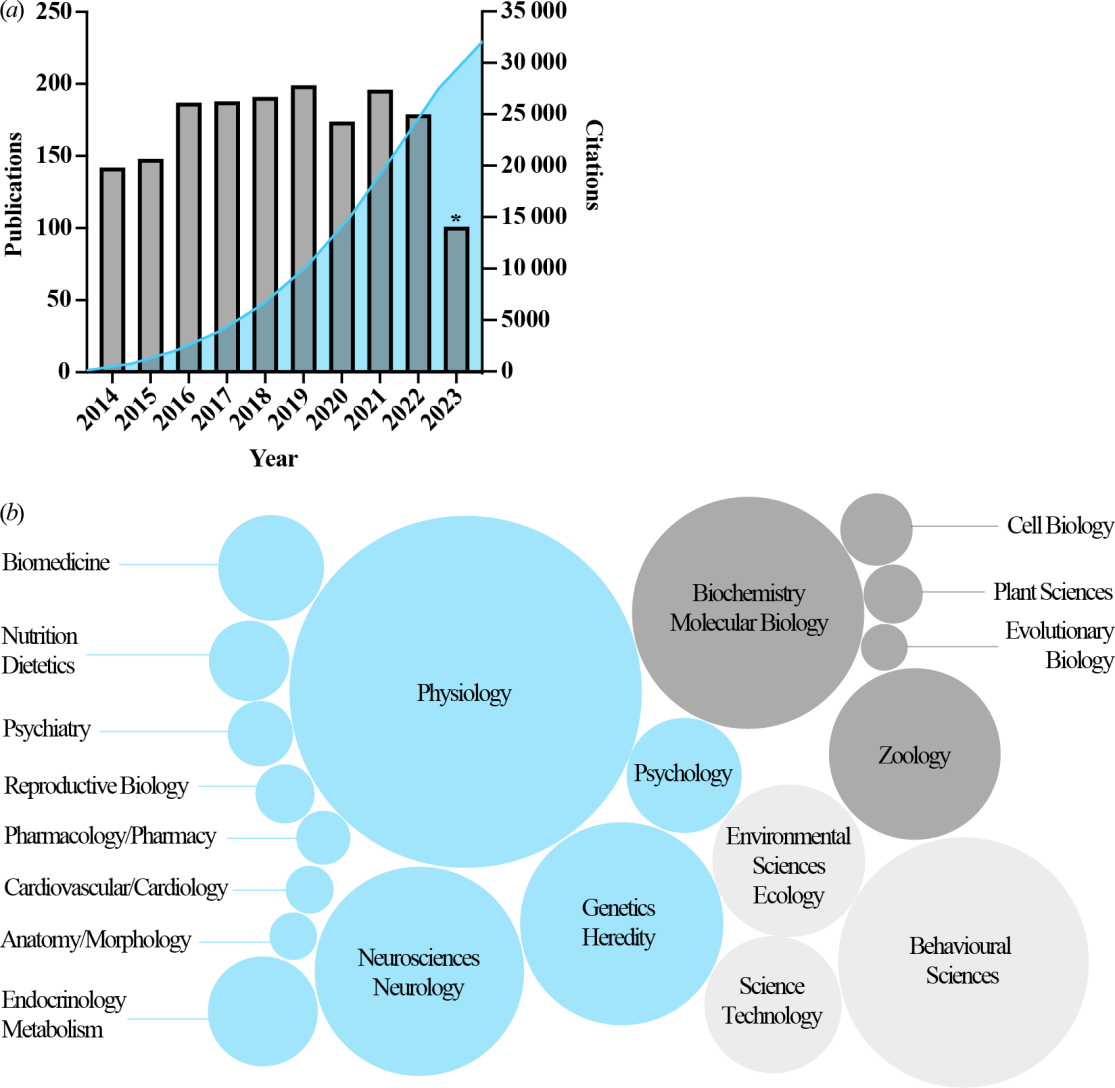
Growth of UK chronobiology. The Web of Science database was used to examine the number of publications and citations for the keyword search term ‘circadian’ and affiliation ‘United Kingdom’. For the period ranging from 2014 to 2023 a total of 1704 publications were identified and found to be cited 32 086 times. (*a*) Data are plotted as yearly publications (grey) and cumulative citations (blue). UK chronobiology shows a consistent level of annual publications and there has been an exponential increase in the number of citations. Asterisk, data include only the first half of 2023. (*b*) Single variable bubble diagram showing the top 20 research areas associated with the 1704 publications. Size of bubble reflects number of publications associated with that area, with some publications associated with more than one area. Research domains are broadly categorized as ‘health-related’ (blue), ‘biological sciences’ (medium grey) and ‘other’ (light grey). These analyses derived from a search using the term ‘circadian’, which we believe to be (reasonably) diagnostic for circadian chronobiology studies. Where studies address other time scales, or conduct research under non-constant conditions, other terminology such as ‘daily’, ‘diurnal’, ‘ultradian’ and ‘seasonal’ may be used. Our analysis, therefore, underestimates the scale of publications and citations from the UK chronobiology community.

The UK has a thriving chronobiology research community. Between 1967 and 2023, UK-affiliated researchers have published 3200 indexed articles on circadian rhythms, approximately 1704 of them in the past 10 years. The period 2014–2023 has seen a healthy annual publication output, with an exponential citation profile (figure 1*a*). Each publication has received a mean of 18.8 citations, comprising 32 086 total citations over the past 10 years. Web of Science Clarivate analyses indicates that the top 20 research areas covered by these publications range from genetics and cell biology, to behavioural and plant sciences (figure 1*b*). Funding for chronobiology research totalled approximately £160 million of project and PhD training awards active from 2012 to 2022 (estimated from the Research Professional resource, and community surveying). The Medical Research Council (MRC) and Biotechnology and Biological Sciences Research Council (BBSRC) were the equal lead funders, followed by Wellcome and the European Union, with these four funders accounting for £150 million. Additional funds allocated through the UK’s ‘dual support’ system to universities, which typically pays academic staff salaries, are harder to estimate. The UK has a track record of chronobiology leadership, with senior members of the field being pioneers of the molecular chronobiology revolution that ultimately led to the 2017 Nobel Prize in Medicine or Physiology awarded to three of our US colleagues, Jeff Hall, Michael Rosbash and Mike Young. Furthermore, UK researchers have served as presidents of international chronobiology societies (Society for Research on Biological Rhythms (SRBR) and European Biological Rhythms Society (EBRS)). In the UK, the translation of university-based research for socioeconomic benefit is evaluated by the Research Excellence Framework (REF). One part of the REF involves the documentation through case studies of real-world research impacts. The REF 2014 and 2021 submissions included a range of case studies derived from the considerable investment in chronobiology, with 14 impact case studies in 2014 and 12 in 2021. These included the topics of lighting regimes for human health, aviation safety, drug development, reduction of research animal use and software development. The breadth of these case studies showcases the cross-cutting translational and interdisciplinary potential of UK chronobiology.

### (c) UK Clock Club and BioClocks UK

**Figure 2.**
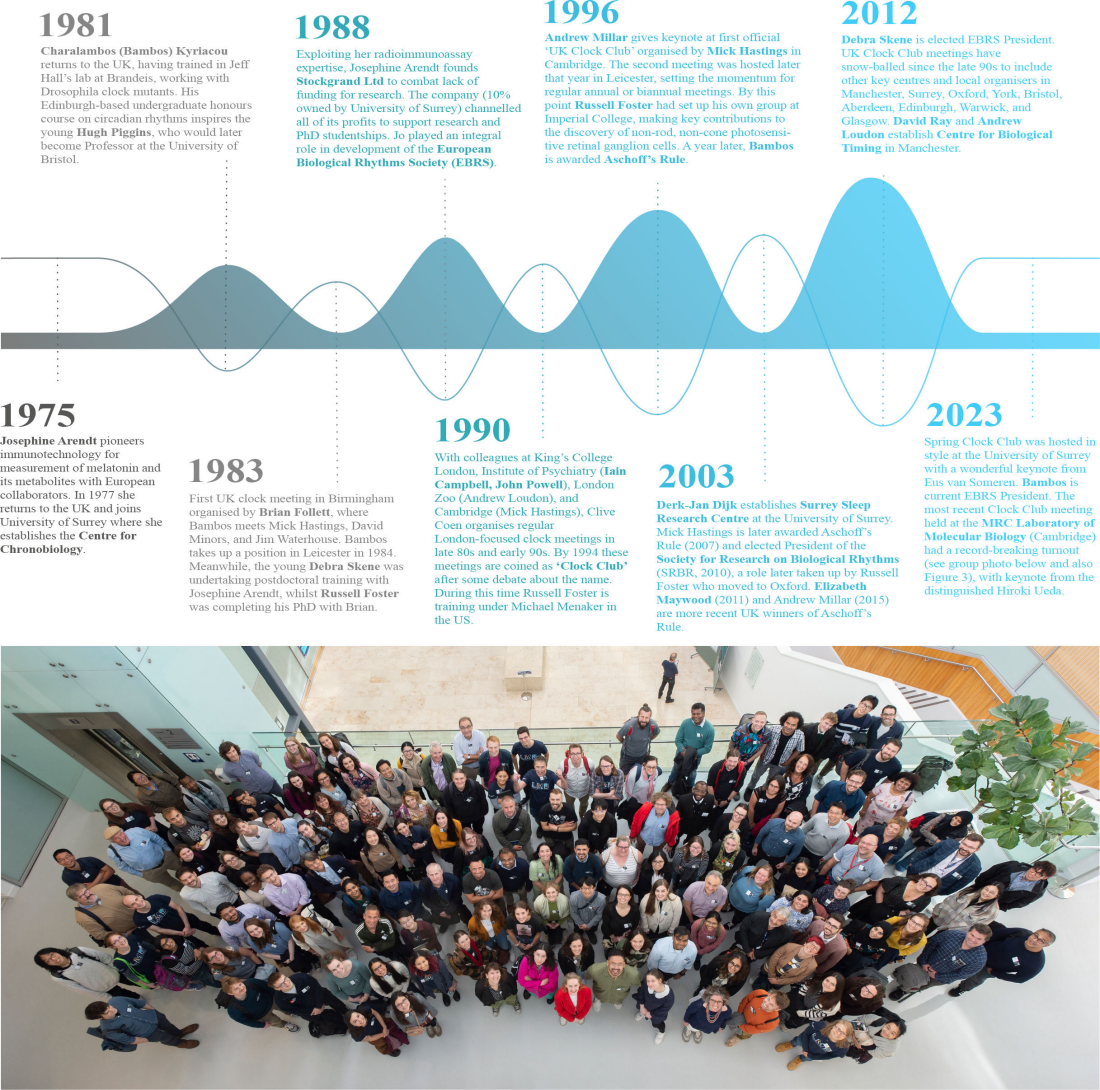
A brief history of the UK Clock Club. For a personal account written by Charalambos (Bambos) Kyriacou, please visit the BioClocks UK website [20]. Please note that this summary and the works cited in this manuscript are not intended to be a comprehensive account of chronobiology in the UK. Many other influential scientists made substantive contributions to the development of this research field. The group photo at Autumn 2023 Clock Club in Cambridge represents around 25% of the current diverse and inclusive community; delegates opting out of photography not shown. Image credit: copyright of the MRC Laboratory of Molecular Biology (LMB), with permission and thanks to Neil Grant and LMB VisLab. Timeline schematic developed using assets from Freepik.com.

**Figure 3.**
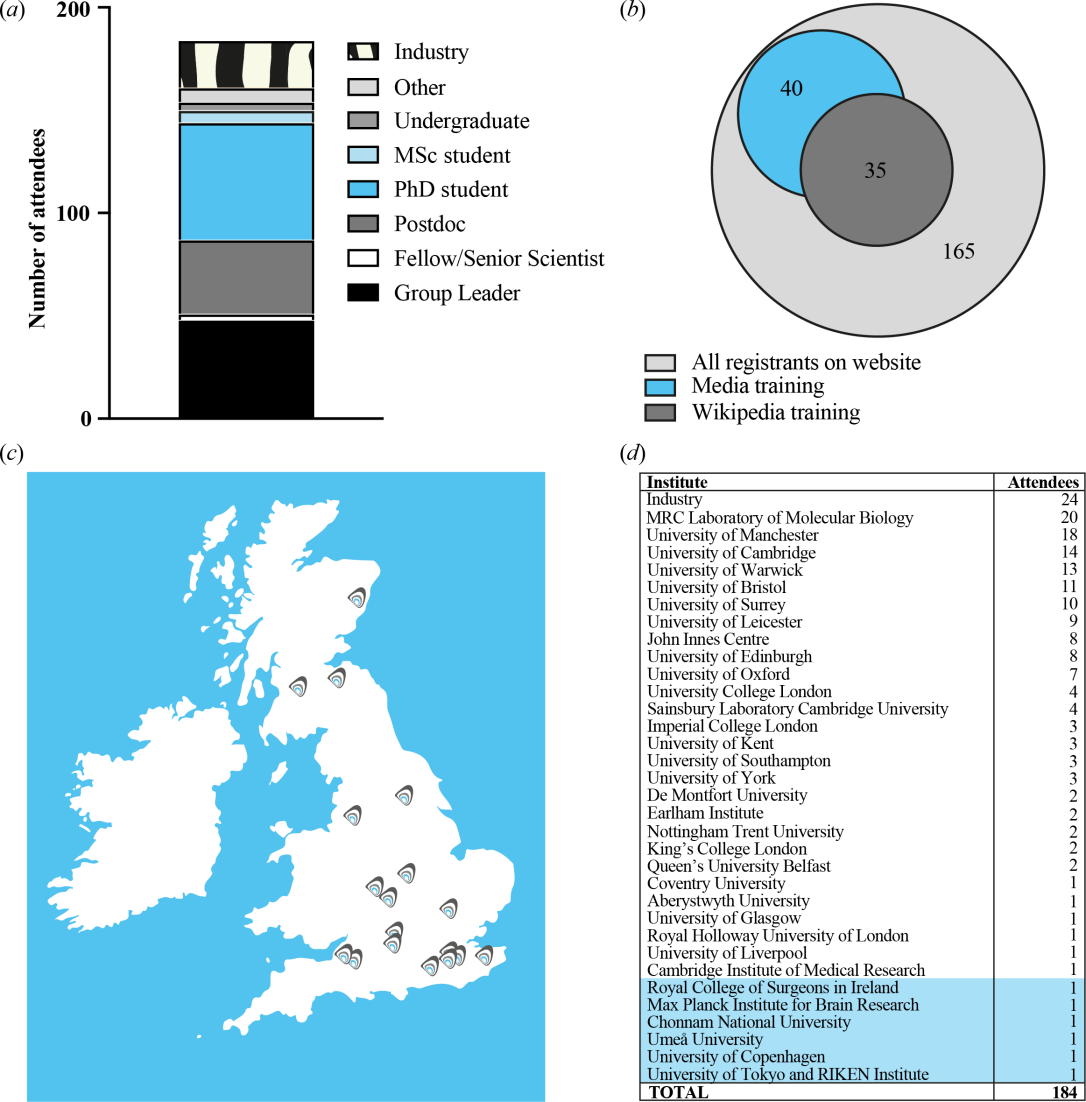
UK Clock Club meetings. Demographics for the latest UK Clock Club meeting (Autumn 2023, MRC Laboratory of Molecular Biology, Cambridge [21]). (*a*) Career stages of 184 registered attendees. (*b*) Proportion of 165 website-registered attendees signing up for BioClocks UK satellite training sessions. Eighteen delegates signed up for both training sessions, and 19 industry sponsor attendees did not register for the meeting via the BioClocks UK website. (*c*) Map showing locations of previous UK Clock Club meetings (chronology of these meetings is shown on the ‘About’ page at the BioClocks UK website [22]). Image credit: copyright of Nina Rzechorzek for BioClocks UK, with permission. (*d*) Delegate numbers for Autumn Clock Club 2023 by institution, with international delegates highlighted in blue.

UK chronobiology would not be where it is today without an active research community brought together by regular (once or twice yearly), free-to-attend, one-day UK Clock Club meetings. The first Clock Club was held in Cambridge in 1996 with around 30 attendees, although there had been sporadic meetings of even fewer clock researchers from the early mid−1980s (figure 2). Since 1996, 44 meetings have occurred across 19 UK academic institutions, and as of 2023, there are 490 registered members of the UK Clock Club mailing list with meetings attracting over 180 attendees (figure 3). The meetings focus almost exclusively on presentations from early career researchers (ECRs), providing a friendly and informal platform for debate and updates on new tools, methods and ideas. They also provide opportunities for networking and stimulate new collaborations. One example from the mid-1980s was the initial encounter of Mick Hastings, a newly minted lecturer in Cambridge with Bambos Kyriacou, a new lecturer in Leicester. They decided to work together on mammalian clock genes, resulting in them providing the first mammalian circadian transcriptomic and proteomic studies [23,24]. They subsequently performed the first molecular analyses of circatidal rhythms in a non-model crustacean [25]. Likewise, in the mid-late 1990s the UK Clock Club helped facilitate a collaboration between a scientist then with little chronobiology experience, Tony Harmar, who with the Clock Club stalwarts, Hugh Piggins, Liz Maywood and Mick Hastings, showed that the VIP receptor was essential for mouse suprachiasmatic nuclei (SCN) mediated circadian rhythms [26]. These examples underscore how the UK Clock Club can stimulate interactions, making it easier to meet in the UK twice a year than at an overseas meeting attended by far fewer UK chronobiologists. Despite travel bursaries and free registration for selected ECRs, international meetings remain unaffordable for many. UK Clock Club is funded by sponsorship and host institutions, and is coordinated by host scientists. Therefore, organizing Clock Club represents a substantial time investment. During the Oxford UK Clock Club in 2022, Andrew Millar pitched the idea of greater community coordination to maximize our impact, and UK Clock Club members were invited to volunteer to help create ‘BioClocks UK’ [22]. Funding proposals to support this vision were refined with community feedback at two online Town Hall meetings and the Clock Club meeting at the University of Surrey (April 2023). Later that year, BioClocks UK received its first funding, from the Royal Society of Edinburgh to establish a research network and, more recently, from BBSRC for impact delivery.

## 2. Translating chronobiology research for real-world impact

Chronobiology has implications for human health and well-being, the productivity of animals and plants, biodiversity and the resilience of our ecosystems. Indeed, circadian clocks are an often underappreciated cornerstone within the ‘One Health’ paradigm, which seeks to understand how the complex and intersecting issues around human and animal diseases, biodiversity loss, agricultural systems and climate change all interact to shape global health. BioClocks UK recognizes the transformative potential for circadian research in informing decisions on healthcare, industrial standards, conservation, farming and agriculture. Below, we consider areas of high-impact value that require urgent attention.

### (a) Health care: disorders and infectious diseases

The circadian clock influences physiology at multiple levels. Medical conditions exhibit time of day variation in symptom expression, from asthma exacerbation [27,28], epileptic seizures [29,30] and inflammatory joint pain [31], to cardiac arrest [32], sudden cardiac death [33], cluster headache [34], migraine [35] and stroke onset [36,37]. Knowledge of the temporal variation of human physiology [38], disease and behaviour can help inform early diagnosis, prognosis and prevention strategies. Moreover, this will underpin efforts to establish appropriate daily timing of interventions for greatest efficacy and safety; indeed, half the targets of widely used drugs are circadian-regulated [39]. Every aspect of brain physiology from proteostasis, to functional activity, blood–brain barrier permeability [40] and even the experience of pain [41] varies over the daily cycle. Chronobiology thus has clear relevance to brain disorders in both acute [42] and chronic [43] settings. Mood also shows a circadian rhythm, and changes in daily activity accompany or even predate episodes of mental illness [44]. Daily and seasonal patterns have been observed for seasonal affective disorder [45], suicide attempts [46,47] and circadian rhythm disruption has been linked to opioid use [48]. Neurodegenerative disorders feature disrupted daily rhythms, sometimes years before clinical onset [49], suggesting causal relationships between sleep and/or circadian disruption and dementia [50]. There is high demand for more fundamental research as well as comprehensive, chronotype-controlled [42] clinical studies to understand the interaction between circadian physiology and brain health [43], and to harness the potential of time-resolved precision medicine. Alongside the expansion of digital health, UK chronobiologists are poised to exploit technological advances enabling high temporal resolution data capture from patients at home and in the clinic.

The roles of circadian clocks in maintaining homeostasis and physiological function extend to the immune system. Key components of the innate and adaptive immune defences are under clock control [24,51]. Infectious diseases can affect circadian rhythms, including sleeping sickness caused by *Trypanosoma brucei* [52] and HIV [53]. Knowledge of whether parasites and pathogens benefit from disrupted host rhythms, and whether they have evolved to weaponize host daily cycles offers new avenues for treating infections [54,55]. For example, medical and veterinary interventions that protect host rhythms (e.g. good sleep hygiene) may be beneficial for reducing infection severity and speeding up recovery. Disentangling the causes and consequences of rhythms in infections is challenging in cases where parasites/pathogens exhibit rhythmicity in their own activities [56,57] and is particularly important when the efficacy of anti-parasite drugs is affected by the parasite’s rhythms [57–59]. Uncovering the mechanisms involved in parasite/pathogen timekeeping offers novel drug/vaccine targets, and understanding the ecology and evolution of parasite rhythms will help make such interventions robust against parasite counter-evolution. For many infectious diseases, the temporal landscape of host–pathogen–vector interactions is changing as a result of anthropogenic forces, yet the epidemiological consequences are unknown. For example, malaria-transmitting mosquitoes are shifting their blood-seeking rhythms towards dawn and dusk to avoid encountering insecticide-treated bed nets, which see widespread use at night [60]. Given that the time of day of infection can determine the severity of many diseases (in part, as a consequence of rhythms in the immune system [61]), altered vector rhythms may have undesirable consequences beyond altering epidemiology.

Together with the Circadian Mental Health network [62] and alongside patient charities and support groups, BioClocks UK is engaging with key stakeholders to promote patient and public involvement, and facilitate priority-setting for research using validated National Institute for Health and Care Research impact pathways [63]. We can further create forums to share chronobiological expertise and best practice with those working in the NHS, care homes, clinical educators, veterinary professionals and the pharmaceutical industry. Furthermore, BioClocks UK is working with epidemiological and evolutionary modellers to predict how infection control interventions alter patterns of disease spread and severity via their impact on the rhythms of hosts, pathogens and vectors.

### (b) Shift-work and social jet lag

In our 24/7 society, an increasing number of people are experiencing social jet lag, whereby biological and social timing become mismatched via the demands of modern life. Social jet lag is correlated with obesity [64], diabetes [65,66], cardiac function [67,68], cognitive performance [69,70], depression [71] and anxiety [72,73]. Contributing factors include a lack of exposure to natural light during the day, artificial light at night (ALAN) [74], irregular sleep and eating patterns as well as social and occupational disruption. A study of 65 000 people from central Europe [64] found that 70% of participants have significantly different sleep-wake patterns during free days compared to work days, indicating a large majority of us may be suffering from social jet lag. In addition, around 20% of the working population in Europe and North America undertakes shift work [75], a pattern of working that poses significant challenges to sleep and circadian health [76]. Unsurprisingly, shift workers have an increased risk of cancer, diabetes, cardiovascular disease, viral infection, asthma and dementia [77–84]. Shift work is most common within hospitality and essential services such as healthcare, manufacturing, transport and communications. The contributing factors described for social jet lag are all relevant and likely exacerbated in those who often shift between very different working schedules. More broadly, we consider those that frequently traverse time zones [85] in an occupational context to be ‘shift workers’ and such travel is not just limited to humans [86]. Optimizing the design of hardware and software of the many light-emitting devices we interact with on a daily basis for work and leisure has been evidence-driven by UK chronobiologists [87]. BioClocks UK can engage with unions, policymakers [88] and the Health and Safety Executive (HSE) to ensure the latest chronobiological data are integrated into legislation on working hours. This includes arguing the case for flexible working hours according to chronotype, where feasible, and experimenting with delayed school start times during adolescence, when we experience the most delayed chronotype of our lifetimes.

### (c) Optimizing lighting in the workplace, home and care facilities

We spend approximately 90% of our time indoors [89]; however, workplaces, schools, hospitals and care homes are built to standards emphasizing sufficient lighting for specific tasks rather than mental well-being and circadian health [90]. Positive impacts of daytime light may require enhanced intensity lighting than is commonly specified in architectural design, while evening and night-time light is frequently so bright that it can disrupt sleep, suppress melatonin and change circadian phase [91]. Exposure to natural light during the day has a positive effect on mood [92], sleep quality [93], work productivity [94], school performance [95] and recovery times in hospitals [95]. The impact of light on the circadian clock is dependent on the intensity, duration and spectra, but also the time at which it is presented [96,97]. Correspondingly, exposure to excess light at night is detrimental, shifting circadian clocks and suppressing sleep. While removing all artificial lighting from our homes and workplaces is impractical, there are ways in which chronobiological research can be leveraged to improve buildings, moderate light exposure, or modify our behaviour. Architects and urban designers can increase the amount of natural light we are exposed to through optimizing building orientation and light prospects, as well as installation of large windows, transparent partitions and skylights with mechanisms for adjustment. Meanwhile, lighting and app designers can aim to minimize light at night. Evidence-based advice can also help managers in hospitals and care homes to create programmes for patients and residents, balancing natural daylight and darkness exposure with practical lighting requirements for care. Both national and local government have a role to play in limiting light pollution to ensure healthy rhythmicity and a good night’s sleep. Two workshops held in the UK have played a pivotal role in agreeing expert consensus on new light metrics for predicting chronobiological effects, and then quantifying how much light healthy adults should experience across each 24 hour cycle [91,98]. As those targets start to appear in guidelines for various stakeholders, they motivate a wider consideration of chronobiology in lighting design. Chronobiological research can provide the substrate to innovate appropriate lighting for both public and private spaces.

### (d) Ecology and conservation

Anthropogenic alterations caused by urban living and climate change have wide-reaching effects on the entrainment of biological rhythms, disrupting organisms, populations and ecosystems [99,100]. It is difficult to estimate the true costs of this disruption, but chronobiological research is useful for identifying targets for change. Among examples, ALAN can modify the free-running period length of birds [101], diminish moth caterpillar abundance [102], affect seasonal migrations [102], disrupt sexual signalling in glow worms [103], disturb locomotion activity in marine invertebrates [104], increase coral bleaching [105] and may drive biodiversity loss as nocturnal organisms are put under increased stress [100]. BioClocks UK can support organizations such as DarkSky International, which aims to protect dark skies in parks and reserves (the UK has 7 as of 2013) and educate communities about the importance of dark skies to wildlife. The success of several citizen science projects has revealed the importance of engaging the public in monitoring ALAN and anthropogenic disruption to biological rhythms [106,107].

Invertebrates offer ecosystem services as pollinators, nutrient cyclers and food sources, with almost 35% of invertebrate pollinators currently under threat. Chronobiology can help conserve insect diversity and form the basis for targeted solutions to insect pests, through approaches including the mitigation of the consequences of ALAN on insect behaviour using smart lighting control for particularly sensitive natural environments. Research into how climate change will affect the seasonal cycles of plants and pollinators is important for predicting temporal mismatches between plants and pollinating insects, helping us to identify which relationships are most in need of protection [108,109]. Insect conservationists can also draw on existing research describing the role of insects active at different times of day, to preserve temporal environmental niches [110]. A further example of applying circadian research is in advising on strategies for pesticide use. The use of three neonicotinoid insecticides was banned in the EU in 2018 due to their effect on non-target insects. Neonicotinoids affect circadian regulated processes in bees, causing reduced sleep, disrupted memory formation and changes in behavioural foraging patterns [111,112]. It is crucial that chronobiologists continue to have conversations with wildlife charities, beekeepers, farmers, agrochemical companies and government representatives to convey the importance of circadian and seasonal rhythms in allowing insects to perform their pollinator services required for our food security.

### (e) Agriculture

Chronobiology holds transformative potential for agriculture in improving livestock health, increasing crop yields and climate change resilience, and increasing the efficiency of production. In the UK, approximately 19–23% of cows [113] and 90% of chickens [114] are kept indoors year-round, typically under artificial lighting. Codes of practice from the UK Department for Environment, Food & Rural Affairs (DEFRA) recommend that lighting is bright enough to, ‘clearly see all the housed cattle and for the cattle to feed and behave normally’ [114] and that, ‘animals kept in buildings shall not be kept without an appropriate period of rest from artificial lighting’ [115], yet conditions for optimum circadian health in livestock are not well understood. Light manipulation has multifaceted effects on livestock, such as altered aggressiveness and feather pecking behaviour in laying hens [116], activity levels in broiler hens [117], synchronized circannual rhythms of reproduction in sheep and Japanese quail [118,119], and milk yield and fat content in dairy cows [120,121]. Livestock chronobiology can achieve impact through consultations with g overnment departments (e.g. DEFRA), veterinary professionals (e.g. RCVS/BVA), charities (e.g. RSPCA), food standards companies (e.g. Assured Food Standards) and farming unions (e.g. NFU). This could include photoperiod management and feeding schedules to enhance production [121], timing animal transport to minimize stress [122] and maximizing natural lighting through improved architecture. As research-impact networks evolve, it will be essential to assess whether the circadian-related economic benefits have concurrent benefits for animal welfare, health and climate sustainability.

Circadian clocks underpin the adaptation of crops to seasonal and daily environmental changes. The circadian oscillator genes of several crops (e.g. tomato, soybean) have experienced positive selection during their domestication history, as their global distribution has expanded [2,123,124]. There are two main ways for chronobiology to impact crop production, sometimes coined ‘chronoculture’ [125]. One is to modulate circadian rhythms in crops, to modify crop performance and another is to modify the growth environment to optimize its alignment with the circadian clock. These ideas are discussed in several review articles [125–127]. Modulating circadian rhythms in crops requires knowledge of circadian clock features that confer certain benefits in particular environments. The crops providing most of our calories (wheat, rice and maize) have passed through genetic bottlenecks during breeding, leading to low genetic diversity. It seems likely that circadian characteristics optimized for one location will not apply uniformly over the latitudinal range of cultivation of each crop. This presents opportunities to modify circadian rhythms through conventional breeding and gene editing to increase crop productivity and resilience.

Adaptation of farming practices could include the optimized timing of irrigation or application of herbicides, pesticides and fertilizer to ensure efficient use [128]. The optimal timing of such treatments requires consideration of the circadian rhythms of the crop, as well as the rhythms of the pollinators, pests, soil microbes and plant competitors. Plant chronobiology is relevant to protected and controlled environment (PACE) agricultural systems. These include smart greenhouses, hydroponic systems, vertical farms and speed-breeding platforms. Plant chronobiology is also important post-harvest, with applications in increasing crop nutritional content and food spoilage during transport and retail [129,130]. There is currently a gap between plant and animal chronobiological research and field deployment, which requires research extension to cultivated species, *in situ* field trials and knowledge exchange between chronobiologists, farmers, breeders and policymakers.

### (f) Government policy

The evidence from chronobiological research has the potential to impact many policy areas, as highlighted in the sections above. Chronobiology is directly relevant to recent political debates in the UK including discussions about Secondary school opening hours [131], the effects of artificial light and noise on human health and arguments around abolishing Daylight Saving Time (DST) [132]. DST, advancing the clock by one hour during the spring and reverting in the autumn, was introduced nationally in 1916 to conserve energy for the war effort. Negative consequences of these one-hour shifts in our sleep–wake patterns include higher risks of depression [133], road traffic accidents [133] and heart attacks [134]. Chronobiology research communities have organized consensus statements on the costs of DST [91,135], and while the social effects of altered timing are profound, mitigations will take time to negotiate, as a recent example illustrates. In 2018, the European Commission acted upon the evidence that annual shifts to and from DST were detrimental [136], launching a consultation that garnered 4.6 million responses and 84% approval for change [137]. However, local Permanent Standard Time was rejected by the Republic of Ireland (Eire), posing an unacceptable separation within the island of Ireland, leading to the stalling of this policy in the UK [138]. The potential benefits to shift-workers are equally enmeshed with other constraints on their working conditions. Bringing evidence to bear on this uncertain policymaking process needs both the visibility of research leaders and the persistence and reach of a community-wide organization, like BioClocks UK. Some of the most senior UK chronobiologists have made career-long commitments alongside their research, engaging in policy both directly and through media work that can shift the public debate [139,140]. A community-wide organization should help to mobilize the materials and the scientifically aware staff who can bring the research consensus to these stakeholders, and to feed back the growing range of research questions that will inform policy implementation.

## 3. BioClocks UK vision

**Figure 4.**
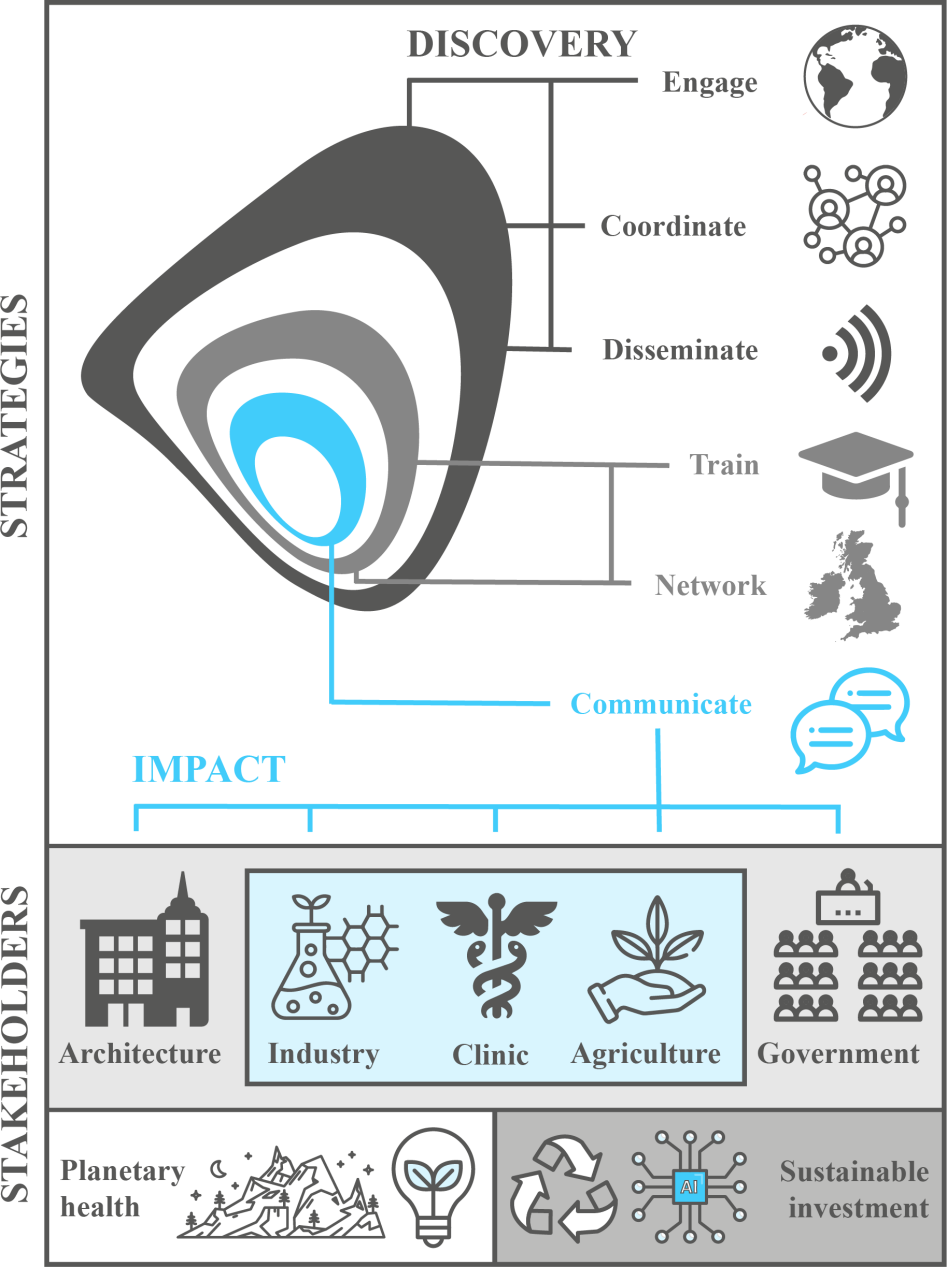
BioClocks UK Vision. Our research network will employ several strategies to deliver impact to key stakeholders. We will *engage* with UK and international sister organizations and facilitate discussions and partnerships to *coordinate* collaborative research efforts across the chronobiology community. We will support chronobiologists to *disseminate* their discoveries to the research community and diverse audiences through multiple media platforms including social media, online resources, podcasts, radio and public events. We will provide opportunities to *train* existing and emerging chronobiology researchers both in field-relevant techniques and transferable skills via symposia and satellite workshops. As part of this training we will procure funding to underwrite events that encourage chronobiologists to *network* with each other and the wider research community (e.g. UK Clock Club meetings), and we will create forums that enable researchers and interest groups to *communicate* bidirectionally for mutual benefit. Stakeholders that can best exploit innovations generated by our community include architecture (construction, town planners, building managers and lighting specialists), industry (pharmaceuticals, bioeconomy/biotechnology, manufacturing, essential services, unions for shift-workers), the clinic (human and veterinary healthcare, public health, clinical research, clinical educators, allied health professionals and paraprofessionals and clinical management teams), agriculture (primary producers of animal and plant products including aquaculture, plant and animal breeders and agri-tech companies) and government (policymakers affecting change in social, education, labour, health and environment sectors). We will embrace and adapt to emerging technologies such as digital health and machine learning, and address under-researched areas such as the chronobiology of marine ecosystems. Overall, our vision is cyclical, with discovery driving impact for stakeholders, and stakeholder needs, solutions and capital driving sustainable research discovery. Icons by Becris, Eucalyp, acongraphic, rawpixel, zirconicusso, Freepik, Muhammad_Usman or developed using assets from Freepik.com.

BioClocks UK was established in 2022; its mission and objectives consolidated input from the chronobiology community, obtained through virtual and in-person surveys and discussions. Objectives of the research network underpin the wider BioClocks UK vision of creating bridges between chronobiology research and real-world impact (figure 4). Some aspects of BioClocks UK were inspired by the past UKRI-BBSRC-funded Genomic Arabidopsis Resource Network (GARNet), which developed UK facilities and platforms to harness the *Arabidopsis* model for plant sciences research. However, the focus of BioClocks UK differs within its aim of delivering impact and policy from chronobiology research.

### (a) Objective 1: communicate with interest groups

BioClocks UK will establish new partnerships with potential interest groups and communicate relevant chronobiological research. Communication is a two-way process, so this objective also involves listening to the public, professionals, industries and policymakers and feeding these questions into research programmes. BioClocks UK will act as a point of contact, bringing scientists together with interest groups and targeting messages relevant to different audiences. The priorities of a member of parliament, healthcare professional, shift worker, pharmaceutical developer or an agri-tech business owner are very different, so communication strategies will reflect this. Effective communication strategies will be built on understanding the views and priorities of target audiences with regards to chronobiology, and how best to represent and share research findings. This objective has four components:

— Develop an understanding of the views and priorities of the target audiences with regards to chronobiology.
— Convey clear messages about the benefits of applying chronobiological research and what actions need to be taken to see these benefits.
— Summarize the most relevant, up-to-date chronobiology research, providing data in an accessible format.
— Provide multiple avenues through which individuals and groups can access this information.

### (b) Objective 2: disseminate material for the network and wider public

BioClocks UK will create content accessible by the general public, and resources for the chronobiology community. This could include radio interviews and podcasts, easily understood material for Wikipedia, YouTube and social media content as well as learning, teaching and outreach resources available through the BioClocks UK website [22]. This new, community-driven website will provide information about tools, events and opportunities for researchers, regular newsletter updates and web-based community forums. A searchable expertise database, accessible through the website, will expand networks, allowing individuals working in media, education or policy to easily identify contacts for chronobiology-related queries. The BioClocks UK website will also provide research guides and toolkits for researchers new to the field, and links to data-repositories and analytical platforms such as Biodare2 [141].

### (c) Objective 3: train researchers and support UK Clock Club meetings

BioClocks UK will coordinate training for chronobiology researchers. An important step in creating impact from chronobiological research is to ensure that scientists have the training they need to make their data accessible, publicize their research findings and communicate confidently with a variety of audiences. Broad training for chronobiology researchers could include rhythmic data analysis and FAIR data management (Findable, Accessible, Interoperable, Reusable) strategies for influencing policy and patient and public outreach [142]. The natural home for some of this training is through break-out workshops within the UK Clock Club meetings, and BioClocks UK will help to support the ongoing success of these meetings into the future.

### (d) Objective 4: coordinate the research network

BioClocks UK will support interactions within the research community and stimulate new areas for collaborative funding. A chronobiology research forum will be created by inviting researchers to participate in strategic workshops, meetings and sandpit events with the twin aims of evolving research topics into themes suitable for future funding and forging new collaborations between researchers. It is important for researchers to get the opportunity to debate topics and review current work in which they have specific expertise, particularly when relevant to policy. BioClocks UK will collate science-informed perspectives and connect researchers through in-person events or through the BioClocks UK website and its forthcoming expertise database.

### (e) Objective 5: engage with international and partner organizations

BioClocks UK will create a formal organization representing the breadth of UK chronobiology that can interact with international chronobiology and sleep organizations. This will synergize global research efforts for greater global impact and help to develop global advocacy strategies. Partnerships will be sought with the European Biological Rhythms Society, the BioClock Consortium (Netherlands), the Japanese Society of Chronobiology and the Society for Research on Biological Rhythms (USA). BioClocks UK will liaise with sister societies to facilitate workshops and webinars of mutual international interest.

### (f) Current action and future prospects

Over the last 50 years, biological timing has grown from an emerging discipline to an accepted component of almost all branches of the life sciences. The UK chronobiology community has a proud history in facilitating that transition. Over the next 50 years, the concepts and discoveries of chronobiology will be increasingly applied for real-world benefit in medicine and health, architecture and planning, working practices and education, government policy and regulation, agriculture and the environment. The vibrant UK chronobiology community is well placed to contribute to that effort via BioClocks UK.

## Data Availability

This article has no additional data.
